# The Double Mortality Burden Among Adults in Addis Ababa, Ethiopia, 2006-2009

**DOI:** 10.5888/pcd9.110142

**Published:** 2012-04-12

**Authors:** Awoke Misganaw, Damen Haile Mariam, Tekebash Araya

**Affiliations:** Addis Ababa Mortality Surveillance Program, College of Health Sciences, Addis Ababa University; College of Health Sciences, Addis Ababa University, Addis Ababa, Ethiopia; College of Health Sciences, Addis Ababa University, Addis Ababa, Ethiopia

## Abstract

**Introduction:**

In Ethiopia, lack of reliable data on causes of death prevents full understanding of the double mortality burden of communicable and noncommunicable diseases. Our objective was to help bridge this research gap by analyzing surveillance data on causes of death in Addis Ababa.

**Methods:**

Burial surveillance identified 58,010 deaths in Addis Ababa from 2006 through 2009, of which 49,309 were eligible for verbal autopsies, a method of interviewing caregivers of the deceased about the circumstances, signs, and symptoms preceding death. We randomly selected 10% from the eligible sample, of whom 91% were defined as adults (aged ≥15 y). Verbal autopsies were completed and causes of death were assigned for 3,709 adults.

**Results:**

Overall, 51% (95% confidence interval [CI], 49.7%-52.9%) of deaths were attributed to noncommunicable diseases, 42% (95% CI, 40.6%-43.8%) to communicable diseases, and 6% (95% CI, 5.5%-7.0%) to injuries. Similar proportions of male and female deaths were caused by noncommunicable and communicable diseases. Adults aged 55 to 84 were more likely, and those aged 15 to 44 were less likely, to die from noncommunicable diseases compared with the age group 85 or older. Premature deaths (defined as earlier than age 65) from noncommunicable diseases were mainly due to certain cancers, type 1 and type 2 diabetes, hypertension, stroke, and genitourinary disease.

**Conclusion:**

Noncommunicable diseases are the leading cause of death among adults in Addis Ababa, where the health care system is still geared toward addressing communicable diseases. Health policy attention is needed to remedy this situation. This observed double mortality burden is unlikely to be unique to Addis Ababa and provides new insight into the epidemiological transition in urban Ethiopia. Nationwide studies should be conducted in Ethiopia to examine the pattern of epidemiological transition and the magnitude of double mortality burden.

## Introduction

For 1,500 years, epidemics of communicable diseases such as plague, cholera, smallpox, typhus, and dysentery were documented as major causes of death in Ethiopia. In the early 20th century, one-fifth of the Addis Ababa population died of influenza (1). In the late 20th century, meningitis, malaria, cholera, and AIDS ravaged the country (2). Analogous data for noncommunicable diseases, however, are scarce (3). Thus, it is difficult to assess the double mortality burden, that is, the increasing threat of noncommunicable diseases as a cause of death while deaths from infectious diseases remain highly prevalent. The health system continues to rely heavily on the conventional infectious disease paradigm and is unresponsive to the emerging epidemiological shift.

Evidence on the causes and patterns of death is required to understand the overall epidemiological profile of diseases in Ethiopia and to help planners and decision makers prioritize the public health agenda. The objective of this study was to examine the proportion of deaths in Addis Ababa caused by communicable diseases, noncommunicable diseases, and injuries using verbal autopsy, a method of interviewing relatives or caregivers of the deceased about their signs, symptoms, lifestyle behaviors, and other characteristics before death and the circumstances surrounding their death.

## Methods

We conducted verbal autopsies of a sample of deceased Addis Ababa residents for whom data were collected by the Addis Ababa Mortality Surveillance Program from September 2006 through December 2009. Our sampling frame was all burials recorded by burial surveillance, which has been conducted since 2001 in all cemeteries within the city limits. Cemetery clerks submitted forms for all deaths (approximately 20,000 per year). In principle, burial surveillance records data for all deceased residents of Addis Ababa, although biases exist because residents may die or be buried outside the capital, just as nonresidents may be buried inside city limits. Some of these biases are identified and corrected, but others inevitably go unnoticed (4-6). However, the 2007 Ethiopian census showed that Addis Ababa had 18,686 deaths of adults (defined for our study as aged ≥15) in the year before the census, a death rate of 9.2 per 1,000 (7). This is similar to the number identified by burial surveillance, 18,013, an age-specific death rate of 8.9 per 1,000. Similarly, adult deaths identified in 2008 and 2009 were 17,984 and 18,154, respectively.

The study population was all adults buried in Addis Ababa cemeteries. Addis Ababa has 89 cemeteries; 64 are Ethiopian Orthodox Church-based, 10 are municipal, 9 are mosque-based, and the rest are associated with the Catholic Church, Greek Orthodox Church, and synagogues. Because cremation is not practiced in Addis Ababa, virtually all deaths result in burials conducted at religious or municipal sites (8).

The burial surveillance identified 58,010 deaths during the study period. Of these, 49,309 (85%) deaths were eligible for verbal autopsy; the rest were not because they had been buried without close relatives or friends who could provide information for verbal autopsy interviews. The verbal autopsy questionnaire was adapted from a standardized verbal autopsy questionnaire from the World Health Organization (WHO) and International Network for the Demographic Evaluation of Populations and Their Health in Developing Countries Network. Three pairs of trained data collectors administered the surveys. They underwent frequent refresher training with strict supervision.

We randomly selected 10% (4,931) of deaths from the eligible burial surveillance sample for verbal autopsy; we used a sample for financial and logistical reasons. Of these, 91% (4,494) were aged 15 or older. We completed verbal autopsies for 3,709 (83%) deaths. All underwent physician review, and underlying causes of death were assigned. Verbal autopsy was not conducted for the rest, 785 (17%) of the sample because some caregivers were unwilling to participate and others were not available, despite repeated visits to their residences.

The age group 15 or older was selected for this study because the prevalence of noncommunicable diseases is rapidly increasing for this age category in developing countries and because the age group 15 to 64 is considered the most economically productive. In addition, adult death rates are an indicator for overall death rates (9). Furthermore, burial surveillance tends to underreport infant deaths (10,11).

We adapted the 2006 *Global Burden of Diseases and Risk Factors* to classify causes of death in our study as follows: Group I (communicable diseases, maternal conditions, and nutritional deficiencies), Group II (noncommunicable causes), and Group III (injuries) (12).

We calculated percentages and proportional death rates using Stata software (StataCorp LP, College Station, Texas). We used a binary logistic regression model to assess associations and significant differences; adjusted odds ratios and 95% confidence intervals were calculated to assess strength of associations.

## Results

Of the 3,709 adults for whom verbal autopsies were completed, approximately half were women and 60% were aged 15 to 64 (Table 1). The median age at death was 55 (range, 15-105).

Overall, 51% of deaths were attributed to noncommunicable diseases, 42% to communicable diseases (of which a negligible percentage were due to maternal and nutritional conditions), and 6% to injuries. The cause of death was ill-defined for 7% of deaths (Table 2). These percentages exceeded 100% because multiple causes could be recorded for 1 death. Proportions of male and female deaths due to noncommunicable and communicable diseases were similar, but more women than men died of both. In contrast, injuries were responsible for 10% of male and 3% of female deaths.

The leading cause of death was cardiovascular disease (CVD) (24%); proportions for hypertension (12%) and stroke (11%) were similar and constituted most of the CVD deaths. The second and third most common causes of death were HIV/AIDS (19%) and tuberculosis (12%). Malignant neoplasm (10%) was the fourth leading cause of death; stomach cancer (2%) and other neoplasms (4%) were the most common neoplasms. Digestive tract diseases caused 9% of all deaths; chronic liver disease accounted for 4%. These 5 leading causes of death accounted for three-fourths of all deaths. The other main causes of death were injury (6%) and type 1 and type 2 diabetes (5%) (Table 2).

### Stratification by sex

The proportions of deaths from cardiovascular causes were comparable by sex, although the pattern varied slightly for the 2 major causes (hypertension and stroke) (Table 2). HIV/AIDS was responsible for a higher proportion of deaths among women, and the tuberculosis was higher for men. Similarly, women more often died of malignant neoplasm than did men. In contrast, digestive tract diseases, injuries, and diabetes caused larger numbers of deaths among men than women. Overall, communicable and noncommunicable diseases caused more deaths among women, and injuries caused more deaths among men.

### Stratification by age

HIV/AIDS and tuberculosis were the leading causes of death for the age group 15 to 54, and CVD and neoplasm were the leading causes of death for people aged 55 or older (Table 3). The third, fourth, and fifth leading causes of death in most age groups were noncommunicable diseases.

The proportion of deaths caused by communicable diseases increased with age among people aged 15 to 34 and decreased after that until age 74. In contrast, the proportion of deaths caused by noncommunicable diseases was lower among people aged 15 to 44 but increased gradually until age 74. Proportions of deaths from injuries were higher among people aged 15 to 34 and decreased gradually after that. In general, deaths from noncommunicable diseases increased with age, and injuries decreased with age (Figure).

**Figure.**
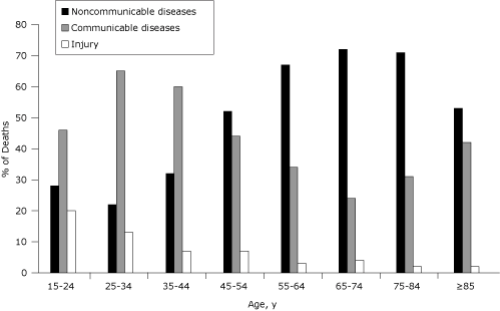
Causes of death in a sample of deceased adults (N = 3,455) in Addis Ababa, by age, 2006-2009. Causes of death may not total 100% because multiple causes could be recorded for 1 death. The cause of death was ill-defined for 254 adults.

**Table:** 

Cause of Death	Age, y							

	15-24	25-34	35-44	45-54	55-64	65-74	75-84	≥85
Noncommunicable diseases, %	30	22	33	52	68	72	71	54
Communicable diseases, %	45	65	60	44	34	25	31	35
Injuries, %	19	13	7	7	3	4	2	2

People aged 55 to 84 were more likely, and people who were aged 15 to 44 were less likely, to die from noncommunicable diseases compared with people aged 85 or older (Table 4).

## Discussion

Most of the deaths in this study were among the young and middle age groups (aged 30-64), reflecting a typical mortality burden for low- and middle-income countries (12). Noncommunicable diseases were the leading cause of death by a considerable margin. Few mortality studies are available for Africa, but this finding is in line with the 2001 estimate for low- and middle-income countries by the *Global Burden of Diseases* analysis, in which 36% of the deaths were due to Group I, 54% to Group II, and 10% to Group III (12). This finding also supports World Bank estimates for Madagascar in 2006, where the percentage of deaths among adults aged 15 to 44 due to Group I and Group II diseases was equivalent (40%) but for adults aged 45 or older was 18% and 63%, respectively (13). Our findings also support those of a study from Banjul, Gambia, where noncommunicable diseases were reported as the leading causes of death among adults (14). These similarities may be due to similar lifestyle and sociodemographic characteristics of urban settings in Africa.

On the other hand, our finding is different from a 2004 WHO estimate for deaths of African adults aged 15 to 59, in which 62% of the deaths were due to Group I diseases, 25% to Group II, and 13% to Group III (15). The World Bank estimate for Kenya in 2006 is also different; 75% of deaths among people aged 15 to 44 were due to Group I, and 11% were due to Group II; 57% of deaths among people aged 45 or older were from Group I, and 27% were from Group II (13).

Moreover, our findings are different from the estimates by the 2002 WHO analysis for Ethiopia (16). In that analysis, noncommunicable diseases accounted for 23% of deaths, and the corresponding estimate for communicable, maternal, perinatal, and nutritional causes was 71%. The possible explanation for these differences could be that cause-of-death analysis by WHO used cause-of-death models because of lack of information on death rates at the country level (17,18). Our results are not directly comparable to a study conducted in rural Ethiopia (19), where leading causes of death are Group I. Our study is from an urban setting dominated by a sedentary, Western lifestyle (eg, cigarette smoking, obesity, stress, and consumption of refined foods).

In our analysis, cardiovascular diseases, HIV/AIDS, tuberculosis, malignant neoplasm, and digestive tract diseases were the leading causes of death and accounted for three-fourths of all adult deaths. This finding supports the indication that an epidemiological transition is occurring, especially in the urban population, while people are also hard hit by HIV/AIDS and tuberculosis (20). An earlier community-based study in Addis Ababa also showed that high blood pressure and physical inactivity were highly prevalent among adults (21). In addition, a study from Zimbabwe, an urban blood pressure follow-up survey, and data from a central registry confirmed high prevalence of hypertension (22).

Our finding that men were more affected by injury concurs with that of a cause-of-death analysis of sub-Saharan Africa (13). Even though our study found that both men and women were almost equally affected by CVD, HIV/AIDS and malignant neoplasm caused more deaths among women than men. Another study found that more women than men die of HIV/AIDS in Addis Ababa (23). The higher proportion of deaths from malignancies among women requires further epidemiological study.

Among young and middle-aged adults, HIV/AIDS, tuberculosis, and injuries were leading causes of death in our study, while CVD, malignancies, and digestive tract diseases were the leading causes among adults aged 45 or older. In general, the proportion of deaths from communicable diseases and injuries decreased with age, while that from noncommunicable diseases increased. This implies that the premature death rate from communicable diseases is higher than that from noncommunicable diseases, a finding that is in line with those of the World Bank's estimates for causes of death in sub-Saharan Africa that reported young adults (aged 15-44) had a high prevalence of HIV/AIDS and high incidence of injuries (13). The pattern of cause of death by age is also in line with the study from South Africa that reported that deaths from communicable diseases decreased with increasing age (24). The contribution of noncommunicable diseases to premature death increases with age starting at age 35. These premature deaths from noncommunicable diseases were mainly due to selected cancers, diabetes, hypertension and stroke.

This study had several limitations. Burial surveillance may not record data for all deceased residents of Addis Ababa, some of whom died outside the city. Verbal autopsies may be less accurate than physical autopsies, and causes of death were ill-defined in some cases.

In conclusion, this study closes some gaps in the estimates of causes of death for Addis Ababa residents that may be relevant to health policy. Noncommunicable diseases are the leading cause of death among adults in Addis Ababa. Together with the existing burden of communicable diseases, this double morality burden requires the attention of policy makers and planners. Leading causes of deaths such as CVD, HIV/AIDS, tuberculosis, malignancies, digestive tract diseases, injuries, and diabetes should be prioritized for attention.

Finally, the observed pattern of double mortality burden is unlikely to be unique to Addis Ababa and provides new insight into the progression of the epidemiological transition in urban Ethiopia. This study may therefore serve as a baseline for further assessments. Similar studies or surveys should be conducted with large cities and rural areas to examine the pattern of epidemiological transition and the factors associated with it.

## Figures and Tables

**Table 1:** Sociodemographic Characteristics in a Sample of Deceased Adults (N = 3,709), Addis Ababa, Ethiopia, 2006-2009

**Characteristic**	Women, n (%)	Men, n (%)
**Age, y**		
15-24	123 (3.3)	94 (2.5)
25-34	270 (7.3)	261 (7.0)
35-44	241 (6.5)	286 (7.7)
45-54	237 (6.4)	250 (6.7)
55-64	234 (6.3)	229 (6.2)
65-74	262 (7.1)	337 (9.1)
75-84	221 (6.0)	248 (6.7)
≥85	257 (6.9)	159 (4.3)
**Religion**		
Orthodox	1,629 (43.9)	1,629(43.5)
Muslim	166 (4.5)	170 (4.6)
Other	50 (1.3)	50 (1.3)
**Ethnicity**		
Amhara	1,001 (27.0)	933 (25.2)
Oromo	453 (12.2)	456 (12.3)
Guraghe	212 (5.7)	248 (6.7)
Other	179 (4.8)	227 (6.1)
**Education status**		
No education	971 (26.2)	484 (13.0)
Elementary school	405 (10.9)	486 (13.1)
Secondary school	276 (7.4)	465 (12.5)
College/university	74 (2.0)	222 (6.0)
Other (church education)	119 (3.2)	207 (5.6)
**Occupation**		
Professional/technical/managerial/sales and services	209 (5.6)	663 (17.9)
Skilled/unskilled manual labor	216 (5.8)	319 (8.6)
Housewives	1,090 (29.4)	8 (0.2)
Retired	79 (2.1)	465 (12.5)
Other (unemployed, students, farmers)	251 (6.8)	409 (11.0)
**Marital status**		
Single	289 (7.8)	515 (13.9)
Married	446 (12.0)	1,032 (27.8)
Separated	133 (3.6)	48 (1.3)
Divorced	139 (3.7)	58 (1.6)
Widowed	838 (22.6)	211 (5.7)

**Table 2:** Causes of Death, by Sex, in a Sample of Deceased Adults (N = 3,709), Addis Ababa, Ethiopia, 2006-2009

Cause of Death	n^a^	% (95% CI)^b^		

		Men	Women	Total
**Communicable diseases, maternal conditions, and nutritional deficiencies**				
**All**	1,565	40 (37.8-42.2)	44 (41.7-46.3)	42 (40.6-43.8 )
HIV/AIDS	707	17 (15.3-18.7)	21 (19.1-22.9)	19 (17.8-20.3)
Tuberculosis	460	14 (12.4-15.6)	11 (9.6-12.3)	12 (11.3-13.5)
Respiratory tract infections	102	3 (2.2-3.8)	3 (2.2-3.8)	3 (2.2-3.3)
Diarrheal disease	115	3 (2.2-3.8)	4 (3.1-4.9)	3 (2.5-3.7)
Meningitis	47	1 (0.5-1.5)	2 (1.4-2.6)	1 (0.7-1.3)
**Noncommunicable diseases**				
**All**	1,904	50 (47.7-52.3)	53 (50.7-55.3)	51 (49.7-52.9)
**Malignant neoplasm**	386	8 (6.8-9.2)	13 (11.5-14.5)	10 (9.4-11.3)
Stomach cancer	73	2 (1.4-2.6)	2 (1.4-2.6)	2 (1.6-2.5)
Breast cancer	43	0	2 (1.4-2.6)	1 (0.7-1.3)
Uterine cancer	47	0	2 (1.4-2.6)	1 (0.7-1.3)
Liver cancer	37	1 (0.5-1.5)	1 (0.6-1.6)	1 (0.7-1.3)
Cancer of cervix	19	0	1 (0.6-1.6)	1 (0.7-1.3)
Colon and rectal cancer	27	1 (0.5-1.5)	1 (0.6-1.6)	1 (0.7-1.3)
Other neoplasm	145	4 (3.1-4.9)	4 (3.1-4.9)	4 (3.3-4.5)
**Diabetes^c^**	183	6 (4.9-7.1)	4 (3.1-4.9)	5 (4.2-5.6)
**Neuropsychiatric conditions**	76	2 (1.4-2.6)	2 (1.4-2.6)	2 (1.6-2.5)
**Cardiovascular diseases**	885	24 (22.1-25.9)	24 (22.1-26.0)	24 (22.5-25.2)
Hypertension	426	11 (9.4-12.4)	12 (10.5-13.5)	12 (10.5-12.5)
Stroke	404	11 (9.4-12.4)	11 (9.6-12.4)	11 (9.9-11.9)
Congestive heart failure	239	6 (4.9-7.1)	7 (5.8-8.2)	6 (5.7-7.2)
Myocardial infarction	84	3 (2.2-3.8)	1 (0.6-1.5)	2 (1.6-2.5)
**Respiratory diseases**	93	2 (1.4-2.6)	3 (2.2-3.8)	3 (2.5-3.7)
Asthma	73	2 (1.4-2.6)	2 (1.4-2.6)	2 (1.6-2.5)
**Digestive diseases**	327	10 (8.6-11.4)	8 (6.8-9.2)	9 (7.9-9.7)
Chronic liver disease	165	5 (4.0-6.0)	4 (3.1-4.9)	4 (3.8-5.1)
Peptic ulcer disease	31	1 (0.5-1.5)	1 (0.6-1.5)	1 (0.7-1.3)
**Genitourinary disease**	137	4 (3.1-4.9)	4 (3.1-4.9)	4 (3.1-4.3)
Chronic renal failure	112	3 (2.2-3.8)	3 (2.2-3.8)	3 (2.5-3.6)
**Injuries**				
**All**	233	10 (8.6-11.4)	3 (2.2-3.8)	6 (5.5-7.0)
**Unintentional**	102	4 (3.1-4.9)	1 (0.6-1.5)	3 (2.2-3.3)
Road traffic accidents	83	3 (2.2-3.8)	1 (0.6-1.5)	2 (1.6-2.5)
**Intentional**	79	3 (2.2-3.8)	1 (0.6-1.5)	2 (1.6-2.5)
Poisoning	28	1 (0.5-1.5)	0	1 (0.7-1.3)
**Ill-defined causes**				
**All**	254	6 (4.9-7.1)	8 (6.8-9.2)	7 (6.0-7.7)

a Values exceeded total deaths because multiple causes could be recorded for 1 death.

b Values above 1 were rounded to nearest whole number and values less than 1 were reported as 0.

c Type 1 and type 2 combined.

**Table 3:** Causes of Death, by Age, in a Sample of Deceased Adults (N = 3,709), Addis Ababa, Ethiopia, 2006-2009

Cause of Death	n^a^	% (95% CI)^b^			

		15-34 y	35-54 y	55-74 y	≥75 y
**Communicable diseases, maternal conditions, and nutritional deficiencies**					
**All**	1,565	59 (55.5-62.5)	53 (49.9-56.1)	29 (26.3-31.7)	33 (30.0-36.1)
HIV/AIDS	707	41 (37.5-44.5)	34 (31.1-36-9)	6 (4.6-7.4)	0
Tuberculosis	460	19 (16.2-21.8)	18 (15.6-20.4)	9 (7.3-10.7)	4 (2.7-5.3)
Respiratory tract infections	102	2 (1.0-3.0)	2 (1.1-2.9)	2 (1.2-2.8)	5 (3.6-6.4)
Diarrheal disease	115	2 (1.0-3.0)	2 (1.1-2.9)	3 (3.0-4.0)	7 (5.3-8.9)
Meningitis	47	3 (1.8-4.2)	2 (1.1-2.9)	0	1 (0.3-1.7)
**Noncommunicable diseases**					
**All**	1,904	25 (21.9-28.1	42 (40.0-45)	70 (67.2-72.8)	63 (59.8-66.2)
**Malignant neoplasm**	386	7 (5.2-8.8)	11 (9.1-12.9)	15 (12.9-17.2)	7 (5.3-8.9)
Stomach cancer	73	1 (0.3-1.7)	1 (0.4-1.6)	3 (3.0-4.0)	2 (1.2-2.9)
Breast cancer	43	0	2 (1.1-2.9)	1 (0.4-1.6)	0
Uterine cancer	47	1 (0.3-1.7)	2 (1.1-2.9)	2 (1.2-2.8)	1 (0.3-1.7)
Liver cancer	37	0	1 (0.4-1.6)	2 (1.2-2.8)	1 (0.3-1.7)
Cancer of cervix	19	0	1 (0.4-1.6)	1 (0.4-1.6)	0
Colon and rectal cancer	27	1 (0.3-1.7)	0	1 (0.4-1.6)	1 (0.3-1.7)
Other neoplasm	145	3 (1.8-4.2)	3 (2.0-4.1)	6 (4.6-7.4)	3 (1.9-4.1)
**Diabetes^c^**	183	1 (0.3-1.7)	4 (2.3-5.2)	8 (6.4-9.6)	6 (4.4-7.6)
**Neuropsychiatric conditions**	76	3 (1.8-4.2)	2 (1.1-2.9)	2 (1.2-2.8)	2 (1.2-2.9)
**Cardiovascular diseases**	885	6 (4.3-7.7)	15 (12.8-17.2)	35 (32.1-37.9)	37 (33.8-40.2)
Hypertension	426	1 (0.3-1.7)	6 (4.5-7.5)	20 (17.6-22.4)	16 (13.6-18.4)
Stroke	404	1 (0.3-1.7)	7 (5.4-8.6)	16 (13.8-18.2)	18 (15.5-20.5)
Congestive heart failure	239	3 (1.8-4.2)	4 (2.3-5.2)	8 (6.4-9.6)	10 (8.0-12.0)
Myocardial infarction	84	0	2 (1.1-2.9)	3 (3.0-4.0)	3 (1.9-4.1)
**Respiratory diseases**	93	1 (0.3-1.7)	2 (1.1-2.9)	4 (2.8-5.2)	3 (1.9-4.1)
Asthma	73	1 (0.3-1.7)	2 (1.1-2.9)	3 (3.0-4.0)	2 (1.2-2.9)
**Digestive diseases**	327	5 (3.4-6.6)	9 (7.2-10.8)	11 (9.1-12.9)	9 (7.1-10.9)
Chronic liver disease	165	2 (1.0-3.0)	5 (3.7-6.3)	7 (5.5-8.5)	3 (1.9-4.1)
Peptic ulcer diseases	31	1 (0.3-1.7)	1 (0.4-1.6)	1 (0.4-1.6)	1 (0.3-1.7)
**Genitourinary diseases**	137	2 (1.0-3.0)	3 (2.0-4.1)	5 (3.7-6.3)	4 (2.7-5.3)
Chronic renal failure	112	2 (1.0-3.0)	3 (2.0-4.1)	4 (2.8-5.2)	3 (1.9-4.1)
**Injuries**					
**All**	233	15 (12.4-17.6)	7 (5.4-8.6)	4 (2.8-5.2)	2 (1.2-2.9)
**Unintentional**	102	6 (4.3-7.7)	3 (2.0-4.1)	2 (1.2-2.8)	1 (0.3-1.7)
Road traffic accidents	83	4 (2.6-5.4)	3 (2.0-4.1)	2 (1.2-2.8)	0
**Intentional**	79	7 (5.2-8.8)	2 (1.1-2.9)	1 (0.4-1.6)	0
Poisoning	28	1 (0.3-1.7)	1 (0.4-1.6)	1 (0.4-1.6)	0
**Ill-defined causes**					
**All**	254	6 (4.3-7.7)	5 (3.7-6.3)	6 (4.6-7.4)	11 (8.9-13.1)

a Values exceeded total deaths because multiple causes could be recorded for 1 death.

b Values above 1 were rounded to nearest whole number, and values less than 1 were reported as 0.

c Type 1 and type 2 combined.

**Table 4:** Odds of Causes of Death in a Sample of Deceased Adults (N = 3,455^a^), Addis Ababa, Ethiopia, 2006-2009

Category	n (%)	Crude OR (95% CI)	*P* Value^b^	Adjusted OR (95% CI)^c^	*P* Value^b^
**Communicable diseases, maternal conditions, and nutritional deficiencies**					
**Age, y**					
15-34	442 (12)	2.70 (2.11-3.46)	<.001	3.63 (2.70-4.88)	<.001
35-44	318 (9)	2.84 (2.18-3.71)	<.001	3.59 (2.66-4.84)	<.001
45-54	214 (6)	1.47 (1.12-1.92)	.006	1.73 (1.30-2.32)	<.001
55-64	156 (4)	0.95 (0.72-1.23)	.72	1.06 (0.80-1.42)	.68
65-74	147 (4)	0.61 (0.46-0.80)	<.001	0.68 (0.51-0.90)	.006
75-84	144 (4)	0.83 (0.63-1.10)	.19	0.90 (0.67-1.19)	.44
≥85	145 (4)	1 [Reference]	NA	1 [Reference]	NA
**Noncommunicable diseases**					
**Age, y**					
15-34	183 (5)	0.28 (0.22-0.36)	<.001	0.20 (0.15-0.27)	<.001
35-44	176 (5)	0.43 (0.33-0.57)	<.001	0.35 (0.26-0.47)	<.001
45-54	253 (7)	0.94 (0.72-1.22)	.62	0.81 (0.61-1.07)	.14
55-64	313 (8)	1.81 (1.37-2.38)	<.001	1.66 (1.25-2.21)	<.001
65-74	433 (12)	2.26 (1.74-2.94)	<.001	2.11 (1.61-2.77)	<.001
75-84	334 (9)	2.14 (1.62-2.83)	<.001	2.07 (1.56-2.74)	<.001
≥85	223 (6)	1 [Reference]	NA	1 [Reference]	NA

Abbreviations: OR, odds ratio; CI, confidence interval; NA, not applicable.

a Ill-defined causes of death (n = 254) were not analyzed.

b Calculated by using χ^2^ test.

c Adjusted for sex, religion, marital status, ethnicity, educational status, and occupation.
